# Personal PM_2.5_ Exposure Using Time-Weighted Average Scenarios in the Seoul Metropolitan Area

**DOI:** 10.3390/toxics14050426

**Published:** 2026-05-12

**Authors:** Jae-Won Choi, Shin-Young Park, Cheol-Min Lee

**Affiliations:** Department of Environmental and Chemical Engineering, Seokyeong University, 14, Seogyeong-ro, Seongbuk-ku, Seoul 12713, Republic of Korea; 2025714006@skuniv.ac.kr (J.-W.C.); tlsdud060900@skuniv.ac.kr (S.-Y.P.)

**Keywords:** particle matter, personal exposure, time-weighted average

## Abstract

Personal exposure assessment is essential in environmental and epidemiological studies. However, conventional methods often do not adequately reflect individuals’ spatiotemporal activity characteristics. This study evaluates the suitability of personal exposure assessment methods using PM_2.5_ as a case study, comparing measured personal exposure concentrations with three exposure estimation scenarios (S1–S3). S1 relies on fixed-site monitoring data, S2 incorporates location-based outdoor concentrations and a single indoor measurement, and S3 integrates individual location with microenvironment-specific concentrations. Using personal PM_2.5_ measurements and time–activity data (TAD) from adults in the Seoul metropolitan area, exposure levels showed substantial variation depending on activity patterns and time spent in different microenvironments. Time-weighted average (TWA)-based estimates differed across scenarios; among them, the one integrating microenvironmental concentrations and TAD showed the closest agreement with measured exposure. In contrast, S1 and S2 generally overestimated exposure. Although S3 slightly underestimated short-term high-concentration events, it showed high correlation (r = 0.78) and low errors (RMSE = 4.79, MAE = 3.70), effectively capturing relative variability in personal exposure. These results suggest that integrating time–activity patterns with microenvironmental concentrations improves the accuracy and reliability of personal exposure assessment and is expected to further enhance the reliability of personal exposure assessment.

## 1. Introduction

Fine particulate matter (PM_2.5_) has an aerodynamic diameter of 2.5 µm or less, allowing it to penetrate deep into the lungs via the respiratory tract; it has been reported to cause respiratory and cardiovascular diseases and is closely associated with increased mortality [1,2,3]. Furthermore, due to its large surface area per unit volume, PM_2.5_ readily adsorbs toxic substances, including heavy metals (e.g., As, Ni, Mn, V, Cr(VI)) and polycyclic aromatic hydrocarbons (PAHs) [4,5,6]. Therefore, accurately assessing PM_2.5_ exposure levels is essential for formulating national-level air quality and environmental health policies.

Epidemiological studies have primarily used monitoring data from outdoor fixed monitoring stations and predictions from air quality and meteorological models, such as the Community Multiscale Air Quality modeling system and the Weather Research and Forecasting model. These models simulate or support the prediction of pollutant concentrations based on emissions and meteorological conditions [7,8], and their outputs have been widely used to assess associations between outdoor PM_2.5_ concentrations and short- and long-term health outcomes, including daily mortality, chronic obstructive pulmonary disease, and lung cancer [2,9,10,11]. However, these approaches primarily reflect ambient outdoor concentrations and do not adequately capture individual-level exposure variability, as individuals spend most of their day indoors [12], and indoor concentrations can vary significantly depending on ventilation, pollution sources, building materials, and occupants’ activities [13,14,15,16]. Therefore, exposure assessments based solely on outdoor concentrations may not adequately reflect actual personal exposure, potentially leading to an underestimation or overestimation of health effects [17,18,19,20,21].

Recent studies have utilized personal monitoring devices to assess personal exposure more precisely [22,23,24]. However, due to discomfort associated with prolonged wear, high costs, and management burdens, their application in long-term follow-up studies of large populations is limited, and they are often restricted to short-term measurements of small groups [25,26,27]. Therefore, practical constraints still exist in assessing long- term personal exposure levels in the general population. Despite these advancements, practical methodologies that can accurately estimate personal exposure while integrating indoor, outdoor, and individual-level variability remain limited. In particular, few studies have quantitatively compared different exposure estimation approaches against measured personal exposure data under actual conditions [28,29].

Therefore, this study was conducted to identify methodologies capable of more accurately assessing personal exposure levels. To this end, PM_2.5_ and PM_10_ concentrations were monitored indoors, outdoors, and at the personal level among adults residing in the Seoul metropolitan area, and three exposure estimation methodologies based on time-weighted averages (TWA) were applied. Furthermore, these results were compared and validated against actual exposure levels measured using personal portable monitors.

## 2. Materials and Methods

### 2.1. Data Collection

#### Study Population and Time–Activity Data

This study used a subset of data from a panel study designed to investigate the association between PM_2.5_ exposure and allergic diseases, including asthma, rhinitis, conjunctivitis, and atopic diseases. The panel study was conducted among adults residing in the metropolitan area, including Seoul and Gyeonggi Province, as part of the Core Technology Development Project for the Prevention and Management of Environment-Related Diseases, supported by the Korea Environmental Industry & Technology Institute (RS-2025-01822971).

In this study, 93 participants with valid personal PM_2.5_ monitoring data, indoor PM_2.5_ measurements, time–activity diary data, and GPS location data were included in the final analysis. Participants with insufficient monitoring data, missing time–activity or location information, or device malfunction were excluded. The main characteristics of the study participants are presented in Table 1. This study received Institutional Review Board (IRB) approval prior to its conduct (IRB No. 2022R0384).

To estimate PM_2.5_ exposure for each study participant, we collected data on indoor and outdoor PM_2.5_ concentrations, Time–Activity Diaries (TADs), and personal location data (latitude and longitude coordinates). TAD and location data were collected using a proprietary application. The TAD was designed to record the microenvironments occupied by study participants (house, office, educational facility, transportation, and other indoor and outdoor) in 30-min intervals. At the same time, personal location data were collected at 1-min intervals to track personal movement paths with high resolution [30].

### 2.2. Collection and Calculation of Indoor and Personal PM_2.5_ Concentration Data

Real-time indoor, outdoor, and personal PM_2.5_ concentration data were collected over a two-year period, focusing on the winter and spring months (November–April), when high PM_2.5_ concentrations are frequently observed [31,32]. The first survey was conducted from 21 November 2022 to 16 April 2023, and the second from 1 November 2023 to 16 April 2024. PM_2.5_ concentrations were measured using a portable monitor based on light-scattering technology; the device specifications used in this study are presented in Table 2. PM_10_ concentrations were collected alongside PM_2.5_ concentrations to serve as input variables for the indoor PM_2.5_ concentration prediction model.

#### 2.2.1. Collection and Calculation of Outdoor PM_2.5_ Concentration Data

**Figure 1:**
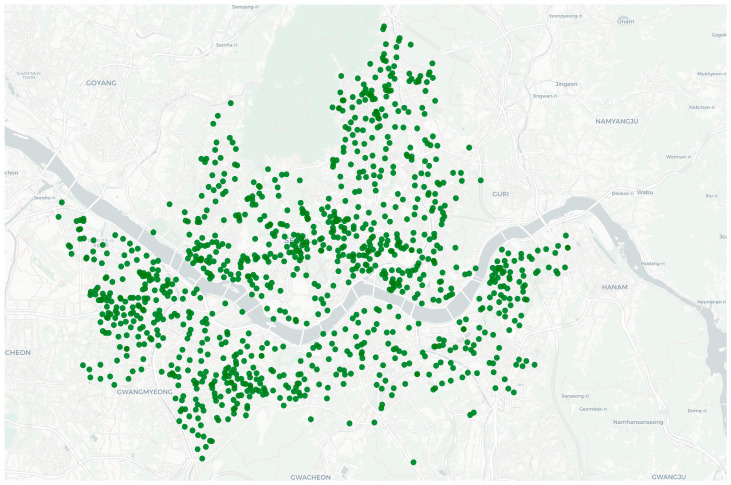
Measurement Locations for Outdoor PM_2.5_ Concentration Measurement [33].

Outdoor PM_2.5_ concentrations were calculated from 1-min-interval PM_2.5_ concentration data collected by approximately 1014 IoT-based optical scattering sensors (OAQ-C300, K-weather, Seoul, Republic of Korea) installed throughout Seoul (Figure 1). The collected data were interpolated to predefined grid-level concentrations with a 500 m × 500 m spatial resolution using Ordinary Kriging (OK). The predicted PM_2.5_ concentrations for each grid cell were combined with the study participants’ GPS location and time data; at each time point, the grid cell corresponding to an individual’s latitude and longitude was identified, and the PM_2.5_ concentration of that grid cell was assigned as the location-based outdoor exposure concentration. Additionally, for outdoor PM_2.5_ concentrations near houses, the grid concentrations corresponding to participants’ residential locations were extracted from the OK interpolation results and used without additional field measurements.

#### 2.2.2. Collection and Calculation of Indoor PM_2.5_ Concentration Data

In this study, PM_2.5_ concentration data were collected across five indoor spaces (house, office, educational facility, transportation, and other indoor and outdoor), accounting for the time individuals spent in each space. Among these, the house is a space where individuals spend approximately 60% of their day [12]; therefore, actual house PM_2.5_ concentrations were measured during the study period. For the remaining indoor microenvironments, where direct measurement was limited, concentrations were estimated using a machine learning-based prediction model [30]. Additionally, depending on the exposure estimation method, both measured and predicted values were used to estimate actual house PM_2.5_ concentrations.

First, PM_2.5_ concentrations in homes were measured by installing IoT-based light-scattering sensors (IAQ-C7, K-weather, Seoul, Republic of Korea) in the living rooms or bedrooms of the 93 study participants’ households. The sensors were installed approximately 1 m away from the floor and walls to minimize the potential impact of indoor airflow and particle diffusion on the measurements [34].

PM_2.5_ concentrations in other microenvironments were calculated using a microenvironment-specific PM_2.5_ concentration prediction model trained via machine learning (AutoML) [35]. This model was configured to predict PM_2.5_ concentrations in the target space on an hourly basis, using six input variables: outdoor PM_2.5_ concentration, outdoor PM_2.5_/PM_10_ ratio, temperature, wind speed, relative humidity, and precipitation. By configuring the model to predict PM_2.5_ concentrations in the target space on an hourly basis, it captured the temporal variability of indoor PM_2.5_ concentrations, as well as temporal changes in personal activity and spatial usage patterns [36,37]. The ExtraTreesRegressor algorithm was applied for house settings, while the XGBRegressor was used for other indoor microenvironments. The outdoor PM_2.5_ and PM_10_ concentrations used as model inputs were derived from OK-interpolated grid data by extracting the values corresponding to the study participants’ location coordinates at each time point.

The machine learning-based prediction model applied in this study was adopted from a previously validated framework, where model performance was evaluated using standard metrics (R^2^, RMSE, MAE) (Table S1), demonstrating reasonable predictive accuracy for indoor PM concentrations [38].

#### 2.2.3. Collection of Personal PM_2.5_ Concentration Data

Personal PM_2.5_ exposure concentrations were measured using a portable monitor (PMM-130, Brilliant & Co., Seoul, Republic of Korea). To ensure a smooth inflow of outdoor air, the monitor was placed in a mesh pouch and worn by the subject. To best reflect the subject’s breathing zone, it was hung at the top of a coat or bag, taking winter wear conditions into account. After providing pre-study training on wearing instructions and precautions, data were collected continuously during daily activities. Detailed information on the personal exposure measurement method and quality control procedures is described in previous studies [30,39].

#### 2.2.4. Calibration of Portable Monitors

Due to their measurement principles, light-scattering-based portable monitors may yield varying readings depending on ambient environmental conditions such as temperature, humidity, and particle characteristics, necessitating on-site calibration [40,41]. Therefore, in this study, for the three types of portable monitors (PMM-130, IAQ-C7, OAQ-C300) used to measure PM_2.5_ concentrations, the high-precision optical particle counter (GRIMM 11-A, GRIMM Aerosol Technik, Germany) was utilized for calibration in previous studies [42,43,44,45] as the reference instrument to perform field calibration through simultaneous measurements in indoor, outdoor, and personal environments. A decision-tree-based multivariate regression model for each concentration interval was applied for calibration [44], and the coefficient of determination (R^2^) relative to the reference instrument after calibration ranged from 0.92 to 0.97. The final calibrated measurements were used as PM_2.5_ concentrations for each space.

### 2.3. Exposure Assessment and Personal Exposure Prediction Scenarios

#### 2.3.1. Methodology for Predicting Personal Exposure Concentrations

In this study, the concept of Time-Weighted Average (TWA) was applied to estimate personal PM_2.5_ exposure concentrations, taking into account individuals’ spatiotemporal movement and activity patterns [46,47,48]. The formula for calculating the daily average concentration based on TWA is shown in Equation (1).

(1) $$C_{TWA}=\frac{\sum_{i=1}^{n}C_{i}\times T_{i}}{\sum_{i=1}^{n}T_{i}}$$

Here, $C_{TWA}$ is the TWA-based daily average concentration (μg/m^3^), $C_{i}$ is the PM_2.5_ concentration (μg/m^3^) at location i and $T_{i}$ is the time (hr) an personal spent at location i. In this study, based on this formula, we estimated personal PM_2.5_ concentration calculation methods and different application methods for occupancy time to estimate personal exposure concentrations. To evaluate the accuracy of the estimated values for each scenario, PM_2.5_ concentrations collected via personal portable monitors were used as the true values of actual personal exposure, and the estimation performance of each scenario was compared and evaluated against this standard. The specific methodologies for the three personal exposure estimation scenarios are as follows.

##### S1—Prediction of Personal PM_2.5_ Concentrations Based on Indoor and Outdoor Fixed Monitoring Station Data

Scenario S1 reflects the fixed-monitoring-station-based exposure estimation methodology commonly used in existing exposure assessment studies [49,50,51]. In this scenario, exposure concentrations were calculated for houses, where people spend most of their time. Indoor concentrations were derived from measured indoor PM_2.5_ concentrations. measurements taken at the study participants’ homes, while outdoor concentrations were calculated using the grid-level concentrations corresponding to the home’s location, interpolated using OK, to estimate personal exposure concentrations using the TWA method.

Time-spent-in-area data were based on the average values presented in the National Institute of Environmental Research’s “The Korean Exposure Factors Handbook” [12]. The formula for calculating the TWA-based daily average concentration for Scenario S1 is shown in Equation (2).

(2) $$C_{TWA\_S1}=\frac{C_{House}\times T_{House}+C_{Outdoor}\times T_{Outdoor}}{T_{House}+T_{Outdoor}}$$

Here, $C_{TWA\_S1}$ is the TWA-based daily average concentration for S1 (μg/m^3^), and $C_{House}$ is the indoor PM_2.5_ concentration in the house (μg/m^3^), $C_{Outdoor}$ is the outdoor PM_2.5_ concentration near the house (μg/m^3^), and $T_{House}$ and $T_{Outdoor}$ are the indoor and outdoor occupancy times (hr), respectively. In this study, the indoor occupancy time was set to 15.86 h, and the outdoor occupancy time was set to 3.34 h.

##### S2—Personal PM_2.5_ Concentration Prediction Reflecting Location-Based Outdoor Concentrations and Measured Indoor Concentrations

Like S1, S2 maintains the spatial framework of indoor and outdoor residential environments but differs in the following two aspects. First, when calculating outdoor exposure concentrations, PM_2.5_ concentrations matched with GPS-based real-time location data ($C_{Outdoor\_GPS}$) were used instead of fixed grid concentrations near the target house ($C_{Outdoor}$) to calculate outdoor exposure concentrations that reflect personal movement patterns. Second, regarding the application of occupancy time, unlike S1, which applied the average values from “The Korean Exposure Factors Handbook” [12], this study used TADs collected from the study participants to apply personal indoor and outdoor occupancy times. This enabled a more realistic TWA-based calculation of personal exposure concentrations that reflect personal lifestyle patterns and movement routes. The formula for calculating the daily average concentration based on TWA in S2 is shown in Equation (3).

(3) $$C_{TWA\_S2}=\frac{C_{House}\times T_{House\_TAD}+C_{Outdoor\_GPS}\times T_{Outdoor\_TAD}}{T_{House\_TAD}+T_{Outdoor\_TAD}}$$

Here, $C_{TWA\_S2}$ is the TWA-based daily average concentration (μg/m^3^) for S2, and $C_{House}$ is Indoor PM_2.5_ concentration (μg/m^3^) in the house.$\;C_{Outdoor\_GPS}$ represents the location-based outdoor PM_2.5_ concentration (μg/m^3^), while $T_{House\_TAD}$ and $T_{Outdoor\_TAD}$ represent the TAD-based personal indoor and outdoor occupancy times (hr) in a house, respectively.

##### S3—Personal PM_2.5_ Concentration Prediction Considering Personal Location and Occupied Space

S3 is a scenario that uses the same location-based outdoor concentrations and personal TADs as in S2, but extends indoor PM_2.5_ concentrations from actual house measurements to microenvironment-specific predictions. In S2, indoor concentration measurements were limited to the household space, making it difficult to measure all microenvironments where individuals spend time; S3 was designed to address this limitation.

In this scenario, personal exposure concentrations were calculated using the TWA method by combining location-based outdoor PM_2.5_ concentrations with microenvironment-specific predicted indoor PM_2.5_ concentrations derived from an outdoor-concentration- based indoor prediction model [30]. The microenvironments were classified using the same five types presented in Section 2.2.2 (house, office, transportation, educational facility, and other indoor), and occupancy time was applied personally based on TAD data collected from the study participants, as in S2. The TWA-based daily average concentration calculation formula for S3 is shown in Equation (4).

(4) $$C_{TWA\_S3}=\frac{\sum_{i=1}^{n}\left(C_{Indoor\_i}\times T_{Indoor\_i,\;\;TAD}\right)+C_{Outdoor\_GPS}\times T_{Outdoor\_TAD}}{\sum_{i=1}^{n}T_{Indoor\_i,\;\;TAD}+T_{Outdoor\_TAD}}$$

Here, $C_{TWA\_S3}$ is the TWA-based daily average concentration (μg/m^3^) for S3, and $C_{Indoor\_i}$ is the predicted indoor PM_2.5_ concentration (μg/m^3^) for microenvironment i. $C_{Outdoor\_GPS}$ represents the location-based outdoor PM_2.5_ concentration (μg/m^3^), and $T_{Indoor\_i,\;TAD}$ and $T_{Outdoor\_TAD}$ are the TAD-based indoor and outdoor occupancy times (hr) for the microenvironment i, respectively.

#### 2.3.2. Calculation of Indoor and Outdoor Exposure Contribution Rates by Scenario

In this study, to identify contribution characteristics at various exposure levels, the indoor and outdoor exposure contribution rates were calculated daily for each scenario and then categorized into two levels: Central Tendency Exposure (CTE) and Reasonable Maximum Exposure (RME). CTE was defined as the indoor and outdoor exposure contribution ratio for the day corresponding to the 50th percentile of the daily TWA concentration distribution, while RME was defined as the indoor and outdoor exposure contribution ratio for the day corresponding to the 95th percentile. The formula for calculating the exposure contribution ratio is given in Equation (5).

(5) $${Contribution}_{i}(\%)=\frac{C_{i}\times T_{i}}{C_{TWA\_S1,\;S2,\;S3}\times 24}\times 100$$

### 2.4. Statistical Analysis

Statistical analysis of the data collected in this study was performed using SPSS Statistics (ver. 23.0, IBM Corp., Armonk, NY, USA). ANOVA and paired *t*-tests were used to compare PM_2.5_ concentrations and time spent in each microenvironment, indoor and outdoor concentrations, and differences between scenario-specific TWA PM_2.5_ concentrations and measured personal exposure concentrations. Additionally, Pearson’s correlation coefficient (r), Mean Absolute Error (MAE), and Root Mean Square Error (RMSE) were calculated to verify the correlation and error between predicted and measured values for each scenario (Equations (6) and (7)). The statistical significance level was set at *p* < 0.05.

(6) $$RMSE=\sqrt{\frac{1}{n}\sum_{i=1}^{n}{\left(y_{i}-{\hat{y}}_{i}\right)}^{2}}$$

(7) $$MAE=\frac{1}{n}\sum_{i=1}^{n}\left|y_{i}-{\hat{y}}_{i}\right|$$

Here, $y_{i}$ is the TWA-based predicted PM_2.5_ concentration for each scenario, and ${\hat{y}}_{i}$ is the measured personal exposure PM_2.5_ concentration. n is the total number of data points.

## 3. Results

### 3.1. Characteristics of Personal PM_2.5_ Exposure Concentrations

#### 3.1.1. Distribution of Personal PM_2.5_ Exposure Concentrations

**Figure 2:**
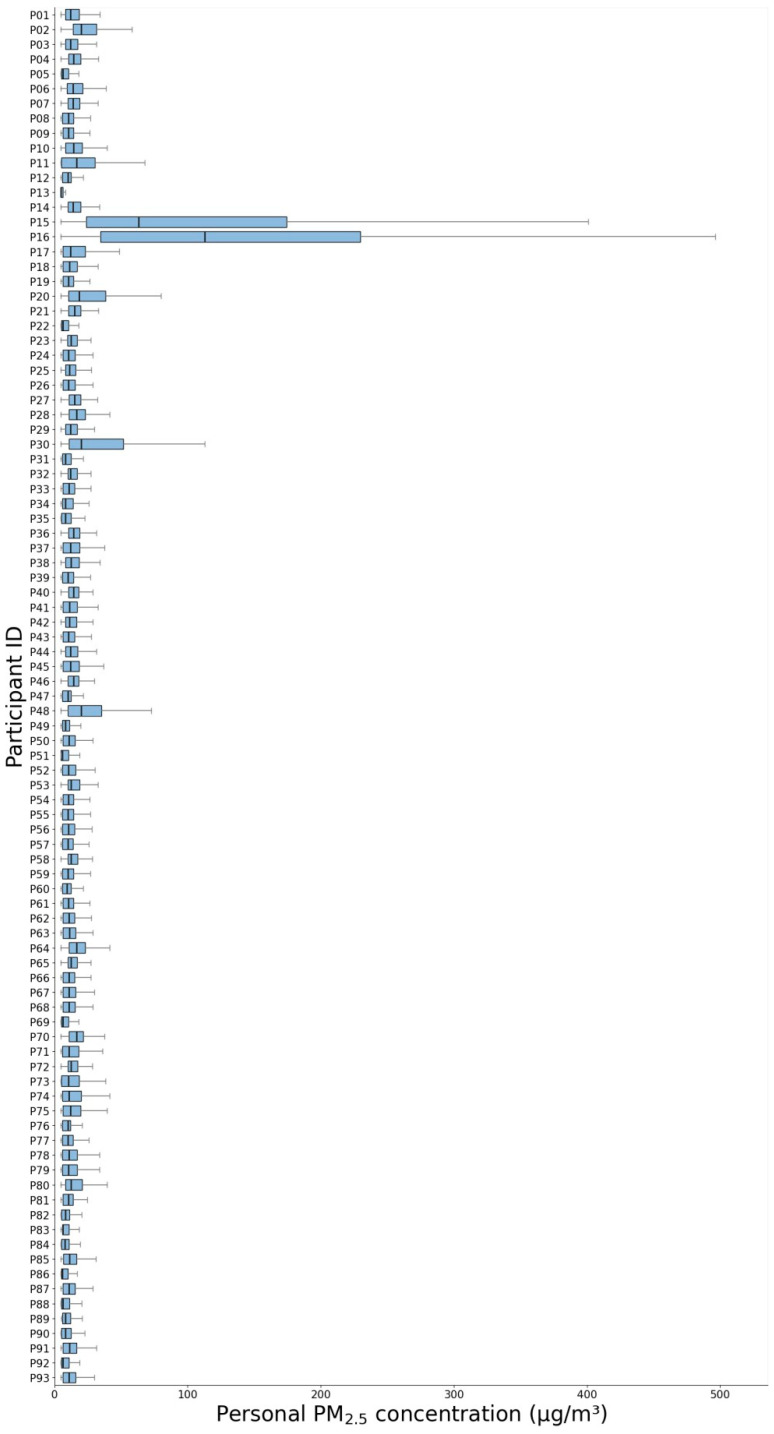
PM_2.5_ Exposure Concentrations by Individual.

The average PM_2.5_ exposure concentration among study participants, measured with a portable personal monitor (PMM-130, Brilliant & Co., Seoul, Republic of Korea), was approximately 12.94 ± 6.66 µg/m^3^. Analysis of the personal exposure distribution (Figure 2) revealed significant variation among participants. Subject P16, who exhibited the highest exposure concentration, recorded 136.58 ± 49.25 µg/m^3^, which was approximately 23.7 times higher than that of subject P13 (5.76 ± 0.94 µg/m^3^). This suggests that PM_2.5_ exposure levels can vary significantly depending on personal lifestyle patterns and living environments, even within the same metropolitan area.

#### 3.1.2. Personal PM_2.5_ Concentrations and Time Spent in Each Microenvironment

Analysis of personal PM_2.5_ concentrations and time spent in each microenvironment (Table 3) revealed that PM_2.5_ concentrations in indoor microenvironments (house, office, educational facility, transportation, and other indoor) were generally similar (11.88–12.30 µg/m^3^, GSD = 1.83–2.10), and no single microenvironment exhibited distinctly higher concentrations. However, considering that individuals spend a relatively longer time in house settings, indoor air quality in house settings is likely to be a significant contributing factor to personal PM_2.5_ exposure.

### 3.2. PM_2.5_ Concentrations by Personal Exposure Prediction Scenario

#### 3.2.1. Comparison of PM_2.5_ Concentration Distributions by Collection Method

Results of the comparison of PM_2.5_ concentrations by indoor and outdoor collection methods (Table 4) showed statistically significant differences in PM_2.5_ concentrations depending on the collection method (*p* < 0.05). Outdoors, location-based concentrations ($C_{Outdoor\_GPS}$, 22.02 ± 9.62 µg/m^3^) were higher than fixed-grid concentrations near residential areas ($C_{Outdoor}$, 15.30 ± 8.88 µg/m^3^) (*p* < 0.05). Indoors, measured concentrations in houses ($C_{House}$, 26.48 ± 9.87 µg/m^3^) were higher than predicted indoor concentrations in houses ($C_{Indoor\_House}$, 13.18 ± 3.76 µg/m^3^) (*p* < 0.05). Meanwhile, predicted indoor concentrations for other microenvironments (office, transportation, educational facility, and indoor) were all lower than those for the house, and there was little variability between spaces. These results suggest that the calculation of personal exposure concentrations may vary depending on the method used to estimate indoor and outdoor concentrations (measured vs. estimated, fixed vs. location-based), thereby directly affecting the accuracy of personal exposure assessments. Additionally, when comparing personal concentrations at home with $C_{House}$, $C_{House}$ were found to be higher than the corresponding personal exposure levels (Figure S1). This indicates that concentration values may vary depending on the measurement method, even within the same indoor environment. Personal exposure concentrations were measured using portable monitors, which reflect individual movement and breathing zone conditions, whereas indoor concentrations were measured at fixed locations, which may be influenced by localized emission sources and limited air mixing. These differences in measurement principles and spatial representativeness may result in systematically higher concentrations in stationary indoor measurements compared to personal exposure levels [52,53,54].

#### 3.2.2. Daily Average TWA PM_2.5_ Concentrations by Personal Exposure Prediction Scenario

A comparison of daily average TWA PM_2.5_ concentrations according to personal exposure prediction scenarios (S1–S3) (Table 5) revealed differences in the concentration levels calculated depending on the scenario. First, for S1, which is based on measured concentrations but does not reflect individuals’ specific activities, the results were approximately 1.5 times higher than actual personal exposure concentrations, indicating an overestimation. Furthermore, S2, which incorporates personal outdoor location data, yielded the highest value at approximately 1.7 times the measured value and exhibited the largest discrepancy from actual exposure concentrations among the three scenarios (RMSE = 19.08, MAE = 12.90). Meanwhile, S3, which combined prediction-based indoor concentrations with temporal activity and spatial coordinate information, showed a value approximately 0.8 times lower than the measured values, indicating a tendency toward slight underestimation; however, with RMSE and MAE of 4.79 and 3.70, respectively—the lowest among the three scenarios—it was confirmed to predict actual personal exposure levels relatively accurately. The CV of actual personal exposure was 35.50%, whereas S1 and S2 showed higher CV values of 44.97% and 46.82%, respectively, indicating that these scenarios tended to overestimate the variability of personal exposure. In contrast, S3 showed a CV of 36.91%, which was the closest to that of actual personal exposure. This suggests that S3 not only reduced prediction errors but also more accurately reproduced the variability of actual personal exposure.

#### 3.2.3. Correlation Analysis by Personal Exposure Prediction Scenario

**Figure 3:**
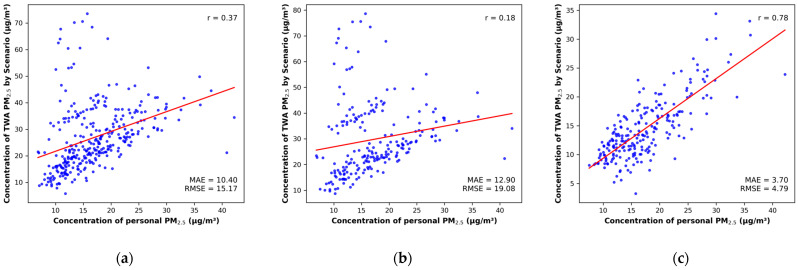
Correlation Between Actual Personal PM_2.5_ Concentration and Predicted TWA PM_2.5_ Concentration; (**a**) S1, (**b**) S2, (**c**) S3.

Analysis of the correlation between measured personal exposure concentrations and scenario-specific TWA concentrations (Figure 3) showed that S3 had the highest correlation coefficient (r = 0.78). In contrast, S1 (r = 0.37) and S2 (r = 0.18) exhibited relatively low correlations. This suggests that S3, which combines predicted indoor concentrations across microenvironments, is effective at reproducing the patterns of actual exposure variability by more accurately reflecting individuals’ spatial movement and occupancy characteristics.

**Figure 4:**
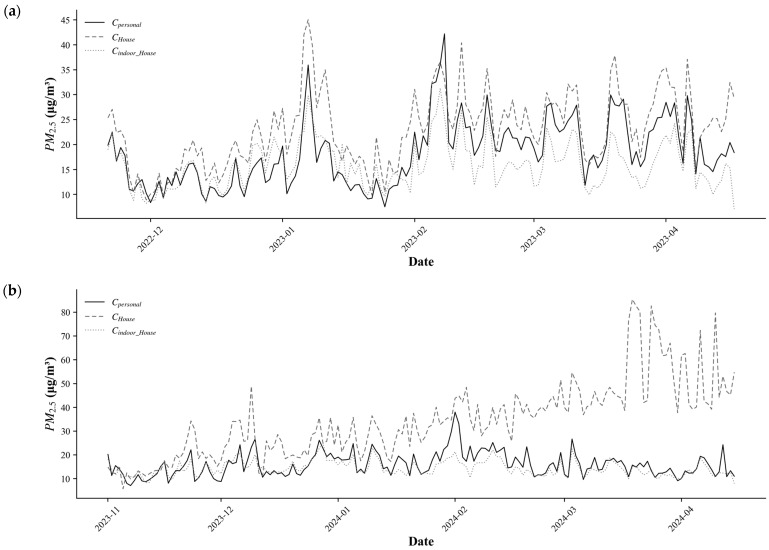
Daily Time-Series Comparison of Personal PM_2.5_.; (**a**) Period 1 (21 November 2022–16 April 2023), (**b**) Period 2 (1 November 2023–16 April 2024).

Meanwhile, the matched daily time-series analysis (Figure 4) showed that with $C_{House}$ were generally elevated during Period 2 (1 November 2023–16 April 2024). S1 and S2, which incorporate $C_{House}$, tended to overestimate these peaks, whereas S3, which is based on $C_{indoor\_House}$, exhibited a smoothing effect. This suggests that differences in peak representation contributed to the outliers observed in Figure 3.

### 3.3. Contribution Rates of Indoor and Outdoor Exposure by Scenario

**Figure 5:**
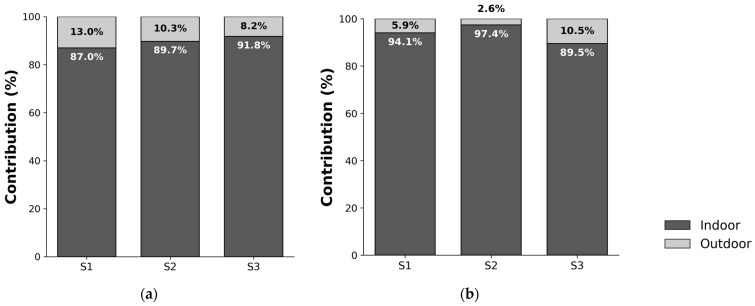
Contribution of Indoor and Outdoor Environments to TWA PM_2.5_ by Scenario; (**a**) CTE (Central Tendency Exposure), (**b**) RME (Reasonable Maximum Exposure).

The results of calculating the contribution rates of indoor and outdoor exposure to TWA concentrations by scenario (Figure 5) showed that, at the CTE level, the indoor contribution rates were S1 (87.0%), S2 (89.7%), and S3 (91.8%). At the RME level, they were S1 (94.1%), S2 (97.4%), and S3 (89.5%), with indoor contributions significantly exceeding outdoor contributions in all scenarios. This suggests that personal PM_2.5_ exposure is primarily determined by indoor environments, where people spend most of their time, and indicates the need to shift from existing epidemiological studies, which have relied on outdoor concentrations as exposure levels, to assessments that incorporate indoor exposure concentrations.

## 4. Discussion

This study was conducted to examine more appropriate methodologies for calculating exposure concentrations to improve the accuracy of personal PM_2.5_ exposure assessments by comparing measured personal PM_2.5_ exposure concentrations with TWA-predicted PM_2.5_ concentrations across three exposure estimation scenarios (S1–S3).

Monitoring of 93 adults residing in the Seoul Metropolitan Area during the study period revealed significant differences in personal PM_2.5_ exposure concentrations among participants. This indicates that exposure levels can vary significantly between individuals, even within the same living area, depending on activity patterns, time spent, and locations visited [55,56,57], supporting the need for exposure assessment methodologies that reflect individuals’ spatiotemporal activity characteristics. Notably, some participants (P15 and P16) showed significantly higher exposure levels, which are presumed to be associated with short-term exposure to specific indoor emission sources or high-concentration microenvironments. However, as the time–activity data were recorded at 30-min intervals, it was not possible to precisely identify the exact sources or conditions responsible for these high exposures.

A comparison of PM_2.5_ concentration collection methods revealed that location-based concentrations were higher outdoors than those from fixed-grid concentrations near house areas. This aligns with previous studies [58] reporting that outdoor concentrations experienced by individuals in urban environments can be higher than those near residential areas. These results are believed to stem from the fact that individuals’ activity spaces in the metropolitan urban environment include areas with various pollution sources, such as commercial districts and traffic-dense areas. Meanwhile, regarding indoor concentrations, measured indoor concentrations in houses were higher than predicted concentrations. Although the house indoor concentration predictions used in this study were designed to reflect average concentration fluctuations by time period, houses serve as primary activity spaces where short-term high-concentration events—such as cooking, smoking, and product use—can occur frequently [59,60,61,62]. While such events can cause abrupt changes in concentration on a minute-by-minute basis, hour-based prediction models may not have adequately captured these short-term fluctuations. Therefore, while prediction-based indoor concentrations can reflect average exposure levels, they may tend to underestimate the high-concentration peaks that occur in actual residential indoor environments, which is interpreted as the cause of the discrepancy with measured concentrations.

A comparison of personal exposure estimation scenarios revealed that S1 and S2 yielded relatively higher RMSE and MAE, indicating larger deviations from actual personal exposure, whereas S3, despite slightly underestimating absolute concentration levels compared to actual personal exposure, demonstrated lower errors (RMSE = 4.79, MAE = 3.70) and reproduced the relative variability in exposure among individuals more accurately. This pattern was further supported by the coefficient of variation (CV), where S3 (36.9%) showed values most comparable to actual personal exposure (35.5%), while S1 (45.0%) and S2 (46.8%) exhibited higher variability, indicating an overestimation of exposure fluctuations. These differences can be attributed to variations in the methods used to estimate indoor and outdoor concentrations, as well as the extent to which individual activities were incorporated in each scenario. In particular, the highest error observed in S2 (RMSE = 24.98, MAE = 19.08) is attributed to the combined influence of location-based outdoor concentrations and measured house indoor concentrations. This can be explained by the overlap of elevated outdoor concentrations and indoor house concentrations in urban environments. These findings are consistent with previous studies [63,64], which reported that outdoor concentrations experienced by individuals in urban environments can be higher than those near residential areas, and that high PM_2.5_ concentrations can also occur indoors. Furthermore, such overlapping inputs may introduce uncertainty and lead to potential overestimation under certain conditions. On the other hand, S3 has the advantage of incorporating both microenvironment-specific predicted concentrations and personal time–activity data, allowing for the consideration of diverse activity spaces. However, it has a limitation in that the indoor prediction model does not sufficiently capture short-term variability during high-concentration episodes. This is consistent with previous studies [30,65], and suggests the need for the development and application of indoor concentration prediction models capable of capturing such high-concentration events in the future. Overall, as shown in Figure S2, these results indicate that, compared with conventional exposure assessment approaches based on fixed monitoring stations, the approach used in this study—incorporating time–activity data and microenvironment-specific concentrations—provides a more realistic representation of personal exposure. In addition, these findings highlight the importance of careful consideration when integrating multiple exposure inputs in personal exposure assessments, particularly when high-concentration values may be simultaneously reflected.

Analysis of the correlation between measured personal exposure concentrations and scenario-specific TWA concentrations revealed that S3, which directly incorporates measured personal indoor and outdoor occupancy times and the corresponding environmental concentrations, exhibited the highest positive correlation (r = 0.78). This confirms that exposure estimation considering personal time–activity information and exposure levels by microenvironment can accurately reproduce actual personal exposure. Previous studies have also highlighted the need to calculate TWA by combining time–activity data with microenvironmental concentrations, noting that relying solely on single indoor concentrations or fixed-point measurements during actual application may underestimate the variability across microenvironments [62,66]. This suggests that a personalized exposure assessment approach can significantly improve exposure accuracy compared to simple estimation methods based on fixed monitoring station concentrations, and that more precise exposure assessments must consider both indoor environmental factors and personal behavioral characteristics. Additionally, approximately 20% of the data points in S1 and S2 were identified as outliers. As results in the time-series analysis, these deviations can be attributed to short-term high peak values in measured house indoor concentrations. S1 and S2 tended to amplify these peaks, indicating that differences in peak representation contributed to the outliers.

Analysis of exposure contribution rates by space revealed that, compared to the actual indoor contribution rate to personal exposure (94.7%), S3 (91.8%) showed the most similar contribution rate at the CTE level, indicating that it relatively well reflects microenvironmental occupancy characteristics, while S1 (87.0%) and S2 (89.7%) tended to estimate a relatively higher outdoor contribution rate. This demonstrates that indoor spaces make a significant contribution to overall exposure concentrations in personal exposure assessments. Previous studies have also reported that residential interiors account for 80–90% of daily occupancy time and constitute a major microenvironment, explaining 85–93% of total PM_2.5_ exposure [67]. Contribution rates are determined by both concentration levels and time spent in each environment. S2, which relies on a single indoor concentration, may overrepresent high-concentration conditions, leading to potential overestimation. In contrast, S3 incorporates microenvironment-specific concentrations and time–activity data, providing a more balanced representation of exposure variability. Accordingly, even when indoor contribution is relatively lower, S3 shows better agreement with measured personal exposure. Contribution rates are limited as direct indicators of estimation accuracy. However, they remain useful for capturing the role of indoor environments in personal exposure assessment.

These findings emphasize that improving model complexity does not necessarily guarantee better performance, and that the quality and representativeness of input data are more critical than the quantity of variables included. This suggests that an approach integrating concentration and time spent in each microenvironment is useful for personal exposure assessment. Nevertheless, limitations remain. In particular, the relatively small sample size and the high proportion of female participants in this study may limit the generalizability of the findings to the broader adult population. In addition, the placement of the portable monitor, which was attached to a bag or coat, may not perfectly represent breathing-zone concentrations. Although efforts were made to position the device as close as possible to the breathing zone under real-world wearing conditions, some measurement uncertainty may remain due to differences between the sampling location and actual inhalation exposure. Furthermore, limitations persist in capturing short-term high-concentration events and accurately representing exposure across diverse microenvironments. The reliability of exposure assessment is expected to improve with the incorporation of higher-resolution microenvironmental data and more detailed personal activity information in future studies.

## 5. Conclusions

To examine the appropriateness of a PM_2.5_ exposure assessment methodology that reflects individuals’ spatiotemporal activity characteristics, this study compared and analyzed measured personal exposure concentrations with TWA-predicted concentrations across three exposure estimation scenarios (S1–S3).

Analysis of measured personal PM_2.5_ exposure concentrations and temporal activity data confirmed that exposure levels can vary significantly depending on an individual’s activity patterns and residence characteristics, even within the same living area. Consequently, a comparison of TWA-based personal PM_2.5_ exposure concentrations across the three exposure scenarios revealed differences in estimated concentration levels depending on the scenario. In particular, S3, which integrated microenvironmental concentration and temporal activity data, showed levels closest to actual personal exposure, whereas S1 and S2, which relied on fixed monitoring stations or applied a single indoor concentration, tended to overestimate exposure overall. Meanwhile, although S3 was somewhat underestimated due to limitations in fully capturing short-term high-concentration events observed in actual measurements, it reproduced the characteristics of interpersonal exposure variability relatively well, with high correlation with measured values (r = 0.78) and low errors (RMSE = 4.79, MAE = 3.70).

This suggests that an exposure assessment approach that considers both an individual’s time–activity information and microenvironmental concentrations can better reflect actual personal exposure. Therefore, future personal exposure assessments require methodologies that comprehensively reflect various microenvironments and personal activity characteristics, and it is expected that the reliability of exposure assessments can be improved by securing more precise microenvironmental concentration and personal activity data.

## Figures and Tables

**Table 1:** Characteristics of Study Participants.

Characteristics		Value
Sex (*n*, (%))		
	Male	30 (32.3)
	Female	63 (67.7)
Age, (year)		
	Mean	40
	Median (Range)	38 (19–66)

*n*: number of participants.

**Table 2:** Specifications of Measurement Devices Used in This Study.

Specification	PMM-130 (Brilliant & Company Co., Ltd., Seoul, Republic of Korea)	IAQ-C7 (K-Weather Co., Ltd., Seoul, Republic of Korea)	OAQ-C300 (K-Weather Co., Ltd., Seoul, Republic of Korea)
Dimensions	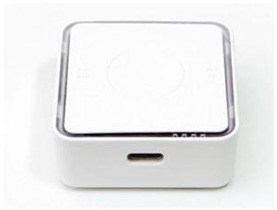 48 (W) × 21 (H) × 48 (D) mm, 0.05 kg	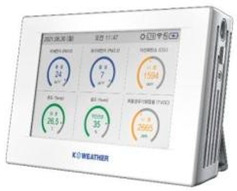 208 (W) × 126 (H) × 53 (D) mm, 0.75 kg	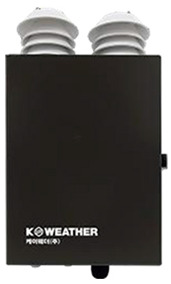 250 (W) × 350 (H) × 150 (D) mm, 5 kg
Metrics	PM_2.5_, PM_10_	PM_2.5_, PM_10_	PM_2.5_, PM_10_
Measurement range		0–1000 µg/m^3^	
Fine dust performance grades ^a^	1st grade (90.0%)	1st grade (90.7%)	1st grade (90.8%)
Flow rate	N/A ^b^	0.1 L/min	1.0 L/min
Operating Temperature	−10 to 60 °C	−5 to 60 °C	−30 to 50 °C
Data storage	Internal memory	Micro SD (Max 32 GB)	Micro SD
Communication	Bluetooth 4.0, Wi-Fi	Wi-Fi, LTE, LTE-M	Wi-Fi, LTE, LTE
Usage	Personal	Home	Outdoor

^a^ Fine dust performance grades refer to the certification grades assigned by the Korean Ministry of Environment based on measurement accuracy, with the values in parentheses indicating the measurement accuracy (%) for each device; ^b^ Not applicable.

**Table 3:** Personal PM_2.5_ concentration (μg/m^3^) and residence time (hr) in microenvironments.

Microenvrionment	PM_2.5_			Residence Time		
			*p*-Value ^c^			*p*-Value ^c^
	GM ^a^	GSD ^b^		Mean	S.D. ^d^	
House	12.29	2.10	*p* < 0.05	11.90	6.20	*p* < 0.05
Office	11.88	1.98		6.43	3.54	
Educational facility	12.02	1.90		2.07	2.08	
Transportation	12.02	1.90		1.24	1.42	
Other Indoor	12.30	1.91		3.68	4.71	
Outdoor	12.93	1.83		1.47	1.80	

^a^ Geometric mean; ^b^ Geometric standard deviation; ^c^ *p*-value from one-way ANOVA; ^d^ Standard deviation.

**Table 4:** Comparison of measured and estimated PM_2.5_ concentrations.

Location		Scenario	Data Type	PM_2.5_(µg/m^3^)		*p*-Value ^b^
				Mean	S.D. ^a^	
Outdoor		S1		15.30	8.88	*p* < 0.05
		S2, S3		22.02	9.62	
Indoor	House	S1, S2		26.48	9.87	*p* < 0.05
		S3		14.56	3.41	
	Office	S3		12.63	4.68	
	Educational facility	S3		12.78	5.08	
	Transportation	S3		13.05	4.56	
	Other Indoor	S3		12.72	4.24	

^a^ Standard deviation; ^b^ *p*-values from paired *t*-test.

**Table 5:** PM_2.5_ Concentration by Personal Exposure Scenario.

Category		Mean	S.D. ^a^	CV ^b^ (%)	Median	Max	MAE ^c^	RMSE ^d^
Personal		16.23	4.87	35.50	16.15	28.35	-	-
Scenario	S1	24.85	9.16	44.97	46.67	10.40	15.17	23.87
	S2	27.11	10.35	46.82	58.96	12.90	19.08	24.98
	S3	13.66	4.45	36.91	24.79	3.70	4.79	13.28

^a^ Standard deviation; ^b^ Coefficient of Variation; ^c^ Mean absolute error (comparing Personal with each scenario); ^d^ Root mean squared error (comparing Personal with each scenario).

## Data Availability

The data used in this study are not publicly available due to institutional restrictions. Access to the data requires permission from the relevant organization. However, the data may be available from the corresponding author upon reasonable request and with permission from the institution.

## Supplementary Materials

The following supporting information can be downloaded at https://www.mdpi.com/article/10.3390/toxics14050426/s1. Figure S1: Comparison of Measured Indoor and Personal PM_2.5_ Concentrations in House Environments; Figure S2: Daily Time-Series Comparison by Scenarios: (a) (21 November 2022–16 April 2023); (**b**) Period 2 (1 November 2023–16 April 2024); Table S1: The results of the MLR model [38].

## Abbreviations

The following abbreviations are used in this manuscript:

PM	Particulate matter
PM_2.5_	Particulate matter with an aerodynamic diameter less than 2.5 µm
PM_10_	Particulate matter with an aerodynamic diameter less than 10 µm
WHO	World Health Organization
NIER	National Institute of Environmental Research
TWA	Time-Weighted Average
IRB	Institutional Review Board
TAD	Time–Activity Data
OK	Ordinary Kriging
AutoML	Automated Machine Learning
CTE	Central Tendency Exposure
RME	Reasonable Maximum Exposure
ANOVA	Analysis of Variance
MAE	Mean Absolute Error
RMSE	Root Mean Square Error
