# Supplementary material for: Personal PM2.5 Exposure Using Time-Weighted Average Scenarios in the Seoul Metropolitan Area
Source: Toxics. 2026 May 12;14(5):426. doi: 10.3390/toxics14050426 (PMC13211426; doi:10.3390/toxics14050426)
Supplement: Supplementary file 1 [file toxics-14-00426-s001.zip › toxics-4257187-supplementary.pdf]

Table S1. The results of the MLR model [38]

Model	N (Train/Test)	Coefficients						Intercept	RMSE	MAE	R^2
		a	b	c	d	e	f				
Previous method	72,300 (50,610/21,690)	16.44	-9.44	4.46	-0.71	-4.57	0.69	9.62	4.86594	3.66157	0.25

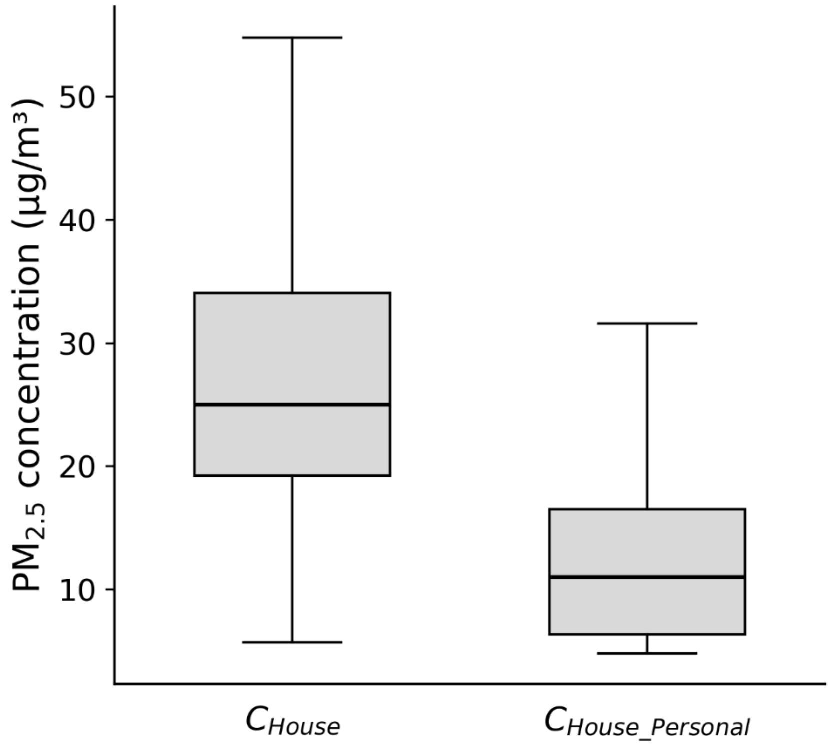

Figure S1. Comparison of Measured Indoor and Personal $PM_{2.5}$ Concentrations in House Environments

(a)

(b)

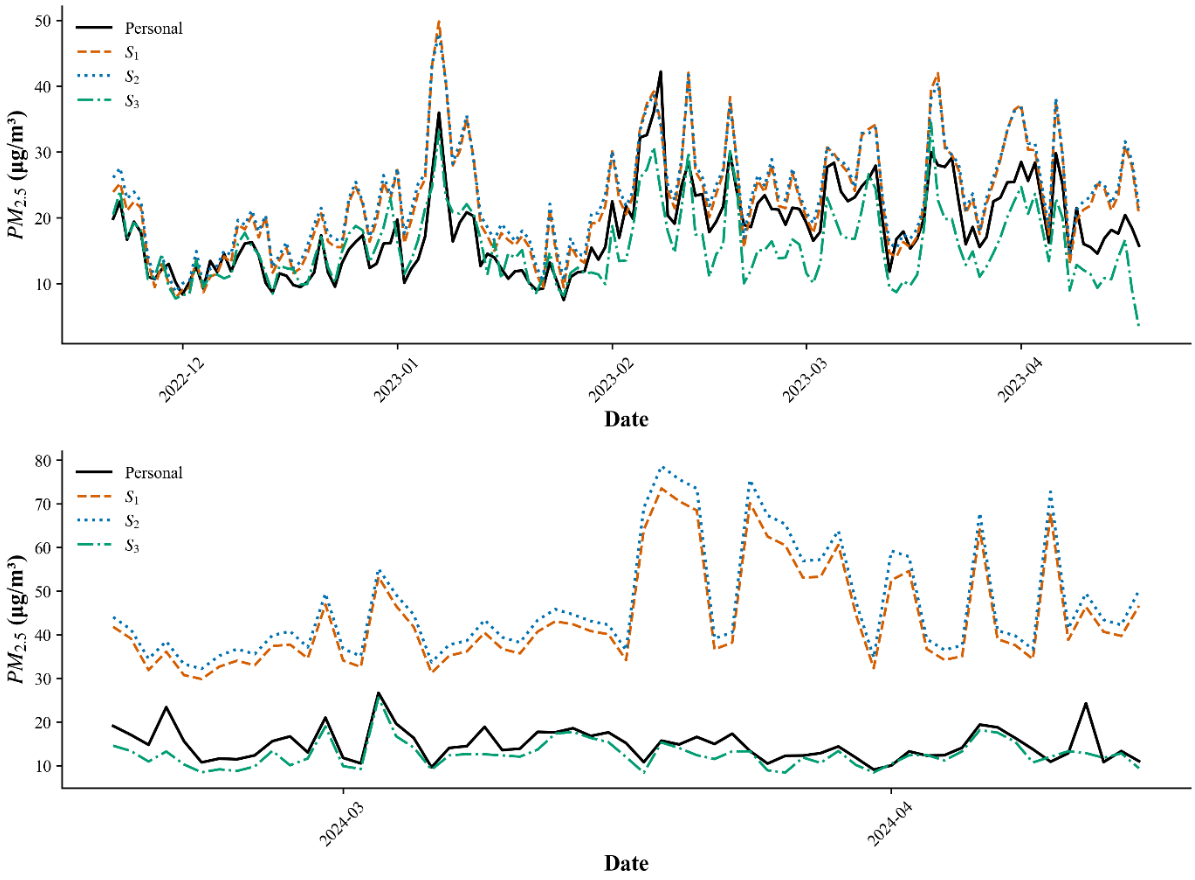

Figure S2. Daily Time-Series Comparison by Scenarios; (a) Period 1 (2022.11.21 – 2023.04.06), (b) Period 2 (2023.11.01 – 2024.04.16)

[38] Park, S.Y.; Yoon, D.K.; Park, S.H.; Jeon, J.I.; Lee, J.M.; Yang, W.H.; Cho, Y.S.; Kwon, J.; Lee, C.M. Proposal of a Methodology for Prediction of Indoor PM2.5 Concentration Using Sensor-Based Residential Environments Monitoring Data and Time-Divided Multiple Linear Regression Model. *Toxics* **2023**, *11*, 526. https://doi.org/10.3390/toxics11060526.
